# Undifferentiated giant cell type carcinoma of the gallbladder with sarcomatoid dedifferentiation: a case report and review of the literature

**DOI:** 10.1186/1752-1947-3-6496

**Published:** 2009-03-18

**Authors:** Andreas Manouras, Michael Genetzakis, Emmanuel E Lagoudianakis, Haridimos Markogiannakis, Artemisia Papadima, George Agrogiannis, Hariklia Gakiopoulou, Panagiotis Kekis, Konstantinos Filis, Efstratios Patsouris

**Affiliations:** 1 First Department of Propaedeutic Surgery, Hippokrateion Hospital, Athens Medical School, University of Athens, Q. Sofias 114 av., 11527 Athens, Greece; 2 Department of Anesthesiology, Hippokrateion Hospital, Q. Sofias 114 av., 11527 Athens, Greece; 3 First Department of Pathology, Athens Medical School, University of Athens, Q. Sofias 114 av., 11527 Athens, Greece

## Abstract

**Introduction:**

Undifferentiated gallbladder carcinoma is a rare entity. Among unusual types of undifferentiated gallbladder carcinoma, giant cell type carcinoma is infrequent and, moreover, very few cases of such neoplasms with osteoclast-like giant cells have been documented. We report a case of undifferentiated gallbladder carcinoma presenting an unusual immunophenotype that was shown to be of giant cell type with sarcomatoid dedifferentiation infiltrated by osteoclast-like multinucleated cells.

**Case presentation:**

An 84-year-old Greek man presented with right upper quadrant pain, high fever, rigors, anorexia and weight loss during the past month. Clinical examination revealed tenderness in the right upper abdominal quadrant and a palpable gallbladder. Blood tests showed elevated white blood-cell count and transaminases. Abdominal ultrasound and computed tomography demonstrated a markedly distended gallbladder, measuring 16 cm x 8 cm, with oedema and pericholecystic fluid, consistent with gallbladder empyema. After an open cholecystectomy and an uneventful recovery, the patient was discharged on the 4^th^ postoperative day. On cut surface, a 2cm solid mass was identified, obstructing the lumen in the neck of the gallbladder. Histopathology and immunohistochemistry offered the diagnosis of an undifferentiated, giant cell type carcinoma of the gallbladder with sarcomatoid dedifferentiation infiltrated with osteoclast-like giant cells.

**Conclusions:**

Undifferentiated, giant cell type carcinoma of the gallbladder with sarcomatoid dedifferentiation infiltrated with osteoclast-like giant cells is a very infrequent neoplasm. Controversy exists over its nature, as related knowledge remains incomplete. Thorough histopathological and immunohistochemical evaluation is imperative for diagnosis. Due to their rarity, the biological behaviour and prognosis of these tumours remain unclear.

## Introduction

The incidence of gallbladder carcinoma is 1.2 cases per 100 000 per year, increasing with age, particularly after the fifth decade of life [1,2]. Risk factors include cholelithiasis, calcified gallbladder wall, adenomatous gallbladder polyps, obesity, oestrogen, choledochal cysts and chemical carcinogens [2]-[4]. Adenoma-carcinoma sequence is highly suspected for gallbladder cancer's aetiology [1,2].

Mainly of epithelial cell origin, the majority of gallbladder carcinomas are adeno-carcinomas of varying degrees of differentiation. Other, less common histological types include clear cell, mucinous, squamous and adenosquamous cell, signet ring cell, small cell, spindle and giant cell, as well as undifferentiated carcinomas with the latter accounting for 10.4% to 10.9% of gallbladder carcinomas [1].

Among undifferentiated gallbladder carcinomas, giant cell type carcinomas may also rarely be encountered; in such cases, it is assumed that anaplastic giant cell components may probably represent dedifferentiation of a pre-existing, well-differentiated adenocarcinoma [2]. Moreover, several types of gallbladder carcinoma may contain a variable number of osteoclast-like giant cells differentiating their biological behaviour as well as prognosis [5,6]. Here, we present a case of undifferentiated gallbladder carcinoma exerting an unusual immunophenotype that was shown to be of giant cell type with sarcomatoid dedifferentiation infiltrated by osteoclast-like multinucleated cells.

## Case presentation

**Figure 1:**
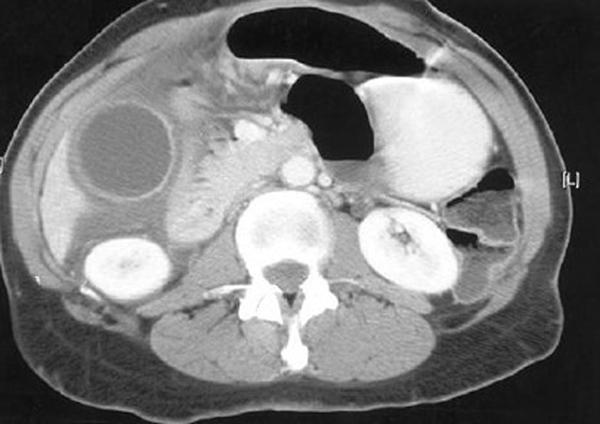
**Abdominal computed tomography scan demonstrated a distended gallbladder with oedema and pericholecystic fluid**.

An 84-year-old Greek man with an unremarkable medical history was admitted to our hospital with right upper quadrant pain, high fever, rigors, anorexia and weight loss over the preceding month. Clinical examination revealed tenderness in the right upper abdominal quadrant and a palpable gallbladder. Blood tests showed elevated white blood cell count and transaminases. Abdominal ultrasound and computed tomography (CT) demonstrated a markedly distended gallbladder, measuring 16 cm x 8 cm, with oedema and pericholecystic fluid consistent with gallbladder empyema (Figure 1). Relying on the patient's clinical picture and the results of the imaging studies, a diagnosis of gallbladder empyema was made. Due to this diagnosis and the big size of the gallbladder, an open cholecystectomy was decided upon. No other abnormality was identified intra-operatively during the assessment of the peritoneal cavity, including lymphadenopathy and liver or peritoneal pathology. Based on the pre-operative as well as the intra-operative findings, open cholecystectomy was performed; no lymph nodes were excised. After an uneventful recovery, the patient was discharged on the 4^th^ postoperative day.

**Figure 2:**
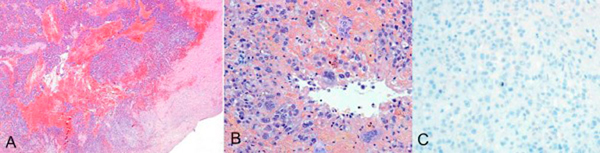
****(A)** Diffuse infiltration by tumour cells**. Invasion of muscularis propria is also present (x20). **(B)** Notice the pleomorphic hypochromatic nuclei with prominent nucleoli, and scattered multinucleated hyperchromatic cells. Haemorrhage and necrotic areas are also present (x200). **(C)** Total immuno-negativity to anti-epithelial membrane antigen (x100).

On cut surface, a 2 cm firm mass obstructing the lumen in the neck of the gallbladder was identified. The mass was solid, yellowish-grey and granular with focal areas of necrosis. Under light microscopy, the tumour demonstrated invasion of the muscularis propria in some areas, with foci of necrosis and haemorrhage (Figure 2A). No extension to the gallbladder serosa was found. Examination of multiple regions under light microscopy did not reveal any vascular invasion. Additionally, no evidence of carcinoma was present in situ.

The neoplastic tissue consisted of diffusely arranged and focally syncytial large and pleomorphic tumour giant cells, with a significant infiltrating population of large multinucleated hyperchromatic cells. The giant tumour cells demonstrated hypochromatic nuclei with prominent nucleoli and severe mitotic activity (Figure 2B). Heterologous elements, such as osteoid and cartilaginous differentiated cells, were not identified. In addition, there was no evidence of any glandular structures. Following haematoxylin-eosin study and according to the morphology of the tumour cells, our differential diagnosis was one of undifferentiated sarcomatoid carcinoma, sarcoma or carcinosarcoma of the gallbladder.

**Figure 3:**
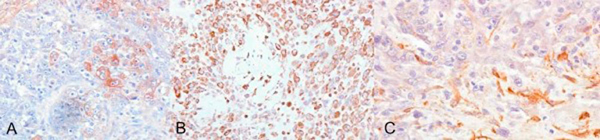
****(A)** Focal immunopositivity to anti-cytokeratin CAM5**.2 (x400). **(B)** Intense staining to anti-vimentin (x100). **(C)** Positively stained tumour cells against anti-smooth-muscle actin (x400).

Immunohistochemistry was performed using commercially available antibodies against epithelial, neuroendocrine and mesenchymal markers. Secondary antibody conjugated with peroxidise-labelled polymer (Envision, Dako Carpinteria,CA, USA) 3' diaminobenzidine was used as chromogen, and finally the slides were lightly counterstained with haematoxylin. The results of immunochemistry stains are presented in Table 1. The tumour demonstrated remarkable immunonegativity to epithelial markers such as epithelial membrane antigen (EMA), carcinoembryonic antigen (CEA), AE1/AE3 and only mild and focal positivity to CAM5.2 (Table 1 and Figures 2C and 3A). On the other hand, mesenchymal markers (vimentin, smooth-muscle actin: SMA) were strongly positive (Table 1, Figures 3B and 3C). The multinucleated cells showed positivity to CD68 stains (Table 1). Osteoclast-like giant cells were considered to be of histiocytic origin because of their PGM1 and KP1 positivity. Markers for neuro-endocrine tumours, lymphomas and melanoma were negative. The entire procedure was repeated by another technician for more reliability, with exactly the same results. Electron microscopy was not performed.

**Table 1:** Results of immunohistochemistry

Antigen	Result
EMA	-
AE1/AE3	-
CEA	-
CAM 5.2	+
CD68 (PGM1)	++
CD68 (KP1)	++
S100	-
Vimentin	+++
SMA	++
F VIII	-
CD4	-
CD34	-
UCHL1	+/-
LYS	-
HMB45	-
CD15	-
CD30	-
CD23	-

(See Abbreviations for full names of the antigens).

Despite the negativity to almost all epithelial markers based on the monophasic tumour immuno-reactivity (that is, there was no evidence of two different components that would have lead to the diagnosis of a carcinosarcoma), the absence of heterologous components, and the focal positivity to CAM5.2 (which is a strong marker for the epithelial origin of a tumour), our final diagnosis was that of a primary, undifferentiated, giant cell type carcinoma of the gallbladder with sarcomatoid dedifferentiation infiltrated with osteoclast-like giant cells.

Following histopathological diagnosis, further thorough investigation did not reveal any abnormality confirming, thus, the diagnosis of a primary gallbladder neoplasm. The patient died 2 months after the operation from disseminated disease; particularly, head, chest and abdominal CT scans showed multiple brain, lung and hepatic metastases.

## Discussion

The patient in this case report presented with a clinical picture of a gallbladder empyema. This clinical diagnosis was furthermore supported by the radiological as well as the intra-operative findings. The diagnosis of gallbladder neoplasm was only made following histopathological evaluation of the resected specimen. The 2 cm solid lesion obstructed the lumen in the neck of the gallbladder, thus leading to the clinical presentation and the imaging and intra-operative findings of gallbladder empyema in our patient. Incidental finding of gallbladder carcinoma following cholecystectomy for symptomatic cholelithiasis, acute cholecystitis, gallbladder empyema or even asymptomatic cholelithiasis has also been observed by others [3]-[5].

Our case proved difficult to classify. The final histopathological diagnosis resulted from a combination of morphology and immunohistochemistry. The morphological characteristics were those of a tumour of epithelial origin. The positive response to CAM5.2, a strong epithelial marker, was an additional characteristic for a carcinoma. According to the World Health Organization (WHO) 2000 classification, undifferentiated carcinoma lacks glandular structures; in the presented case, there was no evidence of such structures, leading to the diagnosis of an undifferentiated gallbladder carcinoma. The neoplastic tissue consisted of diffusely arranged giant cells, with scattered multinucleated cells. The multinucleated cells were morphologically and immunohistochemically compared to those of primary giant cell tumours as well as to osteoclast-like giant cells and were shown to resemble the latter more closely. The unusual feature of the immunophenotype of the presented case is that, despite the epithelioid morphology, the tumour cells demonstrated only focal immuno-positivity to cytokeratins (CAM5.2) and, at the same time, strong positivity to mesenchymal markers (e.g. vimentin). This combination, showing a sarcomatoid dedifferentiation of the tumour, is very rare. A possible explanation for this unusual feature is that keratin expression may decrease, while vimentin expression may increase, as the tumour presents sarcomatoid dedifferentiation. The final diagnosis in our patient was therefore that of undifferentiated gallbladder carcinoma of giant cell type with osteoclast-like giant cells and sarcomatoid dedifferentiation.

Gallbladder carcinomas with osteoclast-like giant cells should be distinguished from giant cell carcinomas. The latter are composed of pleomorphic, undifferentiated giant cells with bizarre nuclei, showing immunohistochemical evidence of epithelial derivation, while displaying an identifiable transition between adenocarcinoma and giant cells [5,6]. However, in our case, there were no demonstrable foci of adenocarcinoma in the tumour. Furthermore, the malignant giant cells were immuno-negative to epithelial staining with only the focal staining with antiCAM5.2 suggestive of their probable histogenesis. CAM5.2 is a keratin mixture, containing a range of cytokeratins between 39 kd and 50 kd, which is a strong marker for the epithelial origin of a tumour.

Moreover, undifferentiated carcinomas with osteoclast-like giant cells are rare, mainly pancreatic and periampulary neoplasms that morphologically mimic giant cell tumours of bones [5,7]. The terminology, histogenesis and biological behaviour of these tumours remain controversial. Several carcinomas may contain a variable number of osteoclast-like giant cells [7]-[12]. In addition, some anaplastic, spindle and giant cell carcinomas of the gallbladder and extrahepatic bile ducts show numerous osteoclast-like giant cells that may simulate giant cell tumours [5]. It is crucial, however, to separate these two types of neoplasm because of striking differences in prognosis. Although prognostic significance of osteoclast-like giant cells is yet to be determined, a more favourable prognosis has been suggested for carcinomas in breast and pancreas [8,13]. However, further studies are required referring specifically to gallbladder cancer.

Epithelial, histiocytic or mesenchymal metaplasia has been suggested to explain the origin of osteoclast-like giant cells. Immunohistochemical analysis has demonstrated the giant cells to be of histiocytic origin lacking of epithelial differentiation. These findings may imply that osteoclast-like giant cells are a specialised form of macrophage [6].

To the best of our knowledge, only 4 cases of osteoclastic-like giant cells infiltrating a gallbladder carcinoma have been reported in the literature so far. The first were presented in 1992 by Grosso et al. [12] and Ito et al. [14], respectively. The neoplasm reported by Grosso et al. was an adenosquamous carcinoma and that by Ito et al. a giant cell tumour. Subsequently, two more cases were reported by Albores-Saavedra et al. [5] and by Akatsu et al. [6], respectively, in 2006, with the latter being an adenosquamous carcinoma. Like our patient, in the case reported by Albores-Saavedra et al., there was also focal cytokeratin positivity to CAM5.2 of the giant cells, and the osteoclast-like giant cells showed immunoreactivity for CD68. Therefore, our case represents the 5^th^ reported case of a gallbladder carcinoma with osteoclast-like giant cells, but the 3^rd^ case of undifferentiated giant cell type and the 1^st^ case exhibiting sarcomatoid dedifferentiation.

## Conclusions

Undifferentiated, giant cell type carcinoma of the gallbladder with sarcomatoid dedifferentiation infiltrated with osteoclast-like giant cells is a very rare neoplasm. Extraskeletal carcinomas exhibiting osteoclast-like giant cells have been suggested to represent a distinct clinicopathological entity with a more favourable prognosis [7]-[9,13]. However, the clinical importance of the phenomenon remains unclear, owing to the rarity of such cases. Our patient died 2 months after the operation from disseminated disease. Poor prognosis could not be associated with the presence of osteoclast-like giant cells, as in our case, the undifferentiated carcinoma showed dedifferentiation with sarcomatoid features probably representing a highly virulent phenotype.

## Abbreviations

AE1/AE3: cytokeratin AE1/AE3; CAM5.2: cytokeratin CAM5.2; CEA: carcinoembryonic antigen; CD68 KP1: KP1 monoclonal antibody to CD68 antigen; CD68 PGM1: PGM1 monoclonal antibody to CD68 antigen; CT: computed tomography; EMA: epithelial membrane antigen; F VIII: factor VIII; HMB45: melanocytic marker - melanoma associated mRNA sequence gp100; SMA: smooth muscle actin; S100: S-100 protein; UCHL1: ubiquitin C-terminal hydrolase 1; WHO: World Health Organization.

## Consent

Written informed consent was obtained from the patient for publication of this case report and any accompanying images. A copy of the written consent is available for review by the Editor-in-Chief of this journal.

## Competing interests

The authors declare that they have no competing interests.

## Authors' contributions

AM carried out the operation and contributed to acquisition of consent and critical review of the manuscript. MG contributed to manuscript conception, research, acquisition of data, drafting and writing of the manuscript. EEL contributed to manuscript conception, research, acquisition of data, drafting and writing of the manuscript. HM contributed to manuscript conception, research, acquisition of data, drafting and writing of the manuscript. AP contributed to the preoperative and postoperative management of the patient and to critical review of the manuscript. GA and HG carried out the histopathologic evaluation and contributed to writing of the manuscript. PK assisted in the operation and contributed to critical review of the manuscript. KF contributed to organising and drafting of the manuscript, and critically revised the manuscript. EP carried out the histopathologic evaluation, contributed to organising and drafting of the manuscript, and critically revised the manuscript. All authors read and approved the final manuscript.
